# Supplementary material for: Excitatory and inhibitory neurochemical markers of anxiety in young females
Source: Dev Cogn Neurosci. 2024 Mar 2;66:101363. doi: 10.1016/j.dcn.2024.101363 (PMC10925933; doi:10.1016/j.dcn.2024.101363)
Supplement: Supplementary file 1 — Supplementary material [file mmc1.docx]

Table S1. **Bayesian Pearson Correlations between age in months and each measure of questionnaire responses for the younger and older age groups.**

|  |  |  |  |  |  |  |  |  | **Prior (medium)** | | |  |  |
| --- | --- | --- | --- | --- | --- | --- | --- | --- | --- | --- | --- | --- | --- |
|  |  |  | **rho** | **95% credible interval** | | **pd (%)** | **% in ROPE** | **Distribution** | **Location** | **Scale** | **BF_10_** | **n** | **Category** |
| Older | Age (Months) | Social anxiety | -0.003 | -0.314 | 0.319 | 50.925 | 44.725 | beta | 3 | 3 | 0.394 | 31 | No evidence |
|  |  | Trait anxiety | -0.019 | -0.346 | 0.306 | 54.275 | 43.925 | beta | 3 | 3 | 0.397 | 31 | No evidence |
|  |  | Depression | -0.199 | -0.476 | 0.126 | 88.775 | 24.375 | beta | 3 | 3 | 0.813 | 31 | No evidence |
| Younger | Age (Months) | Social anxiety | 0.145 | -0.098 | 0.408 | 85.550 | 33.250 | beta | 3 | 3 | 0.564 | 47 | No evidence |
|  |  | Trait anxiety | 0.078 | -0.183 | 0.357 | 71.450 | 45.675 | beta | 3 | 3 | 0.384 | 47 | No evidence |
|  |  | Depression | -0.107 | -0.378 | 0.156 | 78.350 | 41.950 | beta | 3 | 3 | 0.447 | 47 | No evidence |

*Note.* rho is the strength of effect (range -1, 1), signed to indicate direction (positive, negative). 95% credible interval is drawn from the posterior distribution and is the range in which unobserved values can be expected to lie, given the observed data. Pd % is the estimate of the probability of direction (the signed effect, regardless of strength). Pd >95% is indicative of an existing effect. % in ROPE is the percentage of values falling in the null region, or the region of practical equivalence, for correlations this region ranges from -0.05,0.05. The smaller the percentage in ROPE the more confident inferences can be made that the posterior probabilities fall outside of is range, e.g., a measure of significance of the effect. Here, % in ROPE < 2.5 are significant. Prior (medium) refers to the prior distribution selected. Prior is bounded by use of a beta distribution and is defined by a location of 3, and scale 3. BF_10_ refers quantification of evidence in favour of the alternative hypothesis. N is the number of observations in analysis. Category defines the degree of evidence based on BF_10_ value.

Table S2. **Bayesian Pearson Correlations between age in months and each neurochemical in each region.**

|  |  |  |  |  |  |  |  | **Prior (medium)** | | |  |  |  |
| --- | --- | --- | --- | --- | --- | --- | --- | --- | --- | --- | --- | --- | --- |
|  |  |  | **rho** | **95% credible interval** | | **pd (%)** | **% in ROPE** | **Distribution** | **Location** | **Scale** | **BF_10_** | **n** | **Category** |
| ACC | Age (Months) | **Grey matter** | **-0.283** | -0.570 | 0.015 | **95.88** | 12.55 | beta | 3 | 3 | **1.671** | 30 | **Anecdotal** |
|  |  | GABA | -0.109 | -0.422 | 0.227 | 72.63 | 36.80 | beta | 3 | 3 | 0.476 | 30 | No evidence |
|  |  | Glutamate | -0.176 | -0.478 | 0.138 | 84.60 | 26.73 | beta | 3 | 3 | 0.732 | 30 | No evidence |
|  |  | Ratio | 0.018 | -0.311 | 0.347 | 54.45 | 43.35 | beta | 3 | 3 | 0.402 | 30 | No evidence |
| DLPFC | Age (Months) | **Grey matter** | **-0.442** | **-0.618** | **-0.255** | **100** | **0.08** | beta | 3 | 3 | **598.955** | 66 | **Extreme** |
|  |  | **GABA** | **0.387** | **0.165** | **0.564** | **99.98** | **0.53** | beta | 3 | 3 | **72.762** | 66 | **Very strong** |
|  |  | **Glutamate** | **-0.357** | **-0.543** | **-0.147** | **99.95** | **0.73** | beta | 3 | 3 | **31.622** | 66 | **Very strong** |
|  |  | **Ratio** | **-0.417** | **-0.617** | **-0.222** | **99.80** | **0.35** | beta | 3 | 3 | **240.675** | 66 | **Extreme** |
| IOG | Age (Months) | **Grey matter** | **-0.575** | **-0.717** | **-0.406** | **100** | **0.00** | beta | 3 | 3 | **256145.462** | 66 | **Extreme** |
|  |  | GABA | 0.090 | -0.127 | 0.330 | 78.60 | 48.10 | beta | 3 | 3 | 0.383 | 66 | No evidence |
|  |  | **Glutamate** | **-0.326** | **-0.527** | **-0.116** | **99.55** | 2.80 | beta | 3 | 3 | **14.828** | 66 | **Strong** |
|  |  | **Ratio** | **-0.231** | -0.438 | -0.015 | **97.73** | 11.93 | beta | 3 | 3 | 2.023 | 66 | **Anecdotal** |

*Note.* rho is the strength of effect (range -1, 1), signed to indicate direction (positive, negative). 95% credible interval is drawn from the posterior distribution and is the range in which unobserved values can be expected to lie, given the observed data. Pd % is the estimate of the probability of direction (the signed effect, regardless of strength). Pd >95% is indicative of an existing effect. % in ROPE is the percentage of values falling in the null region, or the region of practical equivalence, for correlations this region ranges from -0.05,0.05. The smaller the percentage in ROPE the more confident inferences can be made that the posterior probabilities fall outside of is range, e.g., a measure of significance of the effect. Here, % in ROPE < 2.5 are significant. Prior (medium) refers to the prior distribution selected. Prior is bounded by use of a beta distribution and is defined by a location of 3, and scale 3. BF_10_ refers quantification of evidence in favour of the alternative hypothesis. N is the number of observations in analysis. Category defines the degree of evidence based on BF_10_ value.

Table S3. **Bayesian Pearson Correlations between measures of questionnaire responses and neurochemicals in each region, standardised by sample.**

|  |  |  |  |  |  |  |  | **Prior (medium)** | | | |  |  |
| --- | --- | --- | --- | --- | --- | --- | --- | --- | --- | --- | --- | --- | --- |
|  |  |  | **rho** | **95% credible interval** | | **pd (%)** | **% in ROPE** | **Distribution** | **Location** | **Scale** | **BF10** | **n** | **Category** |
| ACC | Social anxiety | Ratio | 0.095 | -0.254 | 0.392 | 71.50 | 38.80 | beta | 3 | 3 | 0.469 | 30 | No evidence |
|  |  | Glutamate | 0.169 | -0.138 | 0.480 | 84.58 | 28.80 | beta | 3 | 3 | 0.652 | 30 | No evidence |
|  |  | GABA | 0.037 | -0.287 | 0.340 | 57.93 | 43.65 | beta | 3 | 3 | 0.406 | 30 | No evidence |
|  | Trait anxiety | Ratio | 0.243 | -0.071 | 0.538 | 92.38 | 17.85 | beta | 3 | 3 | 1.212 | 30 | Anecdotal |
|  |  | Glutamate | 0.021 | -0.300 | 0.330 | 54.80 | 45.13 | beta | 3 | 3 | 0.401 | 30 | No evidence |
|  |  | GABA | -0.102 | -0.426 | 0.219 | 72.45 | 37.60 | beta | 3 | 3 | 0.481 | 30 | No evidence |
|  | Depression | Ratio | 0.271 | -0.047 | 0.550 | 94.08 | 14.13 | beta | 3 | 3 | 1.442 | 30 | Anecdotal |
|  |  | Glutamate | 0.046 | -0.268 | 0.369 | 60.45 | 42.83 | beta | 3 | 3 | 0.417 | 30 | No evidence |
|  |  | GABA | -0.117 | -0.421 | 0.202 | 75.25 | 36.43 | beta | 3 | 3 | 0.511 | 30 | No evidence |
| DLPFC | **Social anxiety** | **Ratio** | **-0.286** | **-0.472** | **-0.064** | **99.20** | 5.13 | beta | 3 | 3 | **5.758** | 66 | **Moderate** |
|  |  | **Glutamate** | **-0.193** | -0.398 | 0.035 | **95.08** | 19.60 | beta | 3 | 3 | 1.142 | 66 | **Anecdotal** |
|  |  | **GABA** | **0.207** | -0.032 | 0.415 | **95.63** | 18.00 | beta | 3 | 3 | 1.336 | 66 | **Anecdotal** |
|  | **Trait anxiety** | **Ratio** | **-0.263** | **-0.473** | **-0.057** | **98.98** | 7.05 | beta | 3 | 3 | **3.355** | 66 | **Moderate** |
|  |  | Glutamate | -0.091 | -0.316 | 0.138 | 76.88 | 47.93 | beta | 3 | 3 | 0.384 | 66 | No evidence |
|  |  | **GABA** | **0.223** | 0.011 | 0.459 | **96.70** | 13.73 | beta | 3 | 3 | 1.743 | 66 | **Anecdotal** |
|  | Depression | Ratio | -0.190 | -0.405 | 0.051 | 93.85 | 22.73 | beta | 3 | 3 | 0.982 | 66 | No evidence |
|  |  | Glutamate | -0.193 | -0.407 | 0.023 | 95.55 | 18.45 | beta | 3 | 3 | 1.120 | 66 | Anecdotal |
|  |  | GABA | 0.091 | -0.130 | 0.325 | 77.93 | 47.55 | beta | 3 | 3 | 0.386 | 66 | No evidence |
| IOG | Social anxiety | Ratio | -0.157 | -0.371 | 0.075 | 90.70 | 29.85 | beta | 3 | 3 | 0.680 | 66 | No evidence |
|  |  | Glutamate | 0.023 | -0.201 | 0.267 | 57.30 | 58.38 | beta | 3 | 3 | 0.285 | 66 | No evidence |
|  |  | **GABA** | **0.219** | -0.005 | 0.432 | **97.28** | 13.38 | beta | 3 | 3 | 1.670 | 66 | **Anecdotal** |
|  | Trait anxiety | Ratio | -0.042 | -0.256 | 0.198 | 63.28 | 57.95 | beta | 3 | 3 | 0.299 | 66 | No evidence |
|  |  | Glutamate | -0.012 | -0.248 | 0.211 | 54.35 | 58.83 | beta | 3 | 3 | 0.281 | 66 | No evidence |
|  |  | GABA | 0.004 | -0.232 | 0.231 | 51.25 | 60.23 | beta | 3 | 3 | 0.280 | 66 | No evidence |
|  | Depression | Ratio | -0.120 | -0.330 | 0.118 | 84.80 | 39.10 | beta | 3 | 3 | 0.470 | 66 | No evidence |
|  |  | Glutamate | -0.048 | -0.277 | 0.175 | 65.20 | 57.18 | beta | 3 | 3 | 0.301 | 66 | No evidence |
|  |  | GABA | 0.089 | -0.135 | 0.320 | 76.43 | 48.40 | beta | 3 | 3 | 0.372 | 66 | No evidence |

*Note.* rho is the strength of effect (range -1, 1), signed to indicate direction (positive, negative). 95% credible interval is drawn from the posterior distribution and is the range in which unobserved values can be expected to lie, given the observed data. Pd % is the estimate of the probability of direction (the signed effect, regardless of strength). Pd >95% is indicative of an existing effect. % in ROPE is the percentage of values falling in the null region, or the region of practical equivalence, for correlations this region ranges from -0.05,0.05. The smaller the percentage in ROPE the more confident inferences can be made that the posterior probabilities fall outside of is range, e.g., a measure of significance of the effect. Here, % in ROPE < 2.5 are significant. Prior (medium) refers to the prior distribution selected. Prior is bounded by use of a beta distribution and is defined by a location of 3, and scale 3. BF_10_ refers quantification of evidence in favour of the alternative hypothesis. N is the number of observations in the analysis. Category defines the degree of evidence based on BF_10_ value.

Table S4. **Bayesian Partial Pearson Correlations between measures of questionnaire responses and neurochemicals in each region, standardised by sample controlling for Age in months.**

|  |  |  |  |  |  |  |  |  | Prior (median) | |  |  |  |
| --- | --- | --- | --- | --- | --- | --- | --- | --- | --- | --- | --- | --- | --- |
|  |  |  | **rho** | **95% credible interval** | | **pd (%)** | **% in ROPE** | **Distribution** | **Location** | **Scale** | **BF10** | **n** | **Category** |
| ACC | Social anxiety | Ratio | 0.090 | -0.223 | 0.410 | 70.38 | 39.35 | beta | 3 | 3 | 0.469 | 30 | No Evidence |
|  |  | Glutamate | 0.171 | -0.161 | 0.473 | 85.20 | 27.25 | beta | 3 | 3 | 0.680 | 30 | No Evidence |
|  |  | GABA | 0.029 | -0.305 | 0.337 | 57.45 | 43.85 | beta | 3 | 3 | 0.407 | 30 | No Evidence |
|  | Trait anxiety | Ratio | 0.249 | -0.061 | 0.529 | 92.75 | 16.40 | beta | 3 | 3 | 1.216 | 30 | Anecdotal |
|  |  | Glutamate | 0.007 | -0.314 | 0.326 | 51.73 | 45.95 | beta | 3 | 3 | 0.401 | 30 | No Evidence |
|  |  | GABA | -0.101 | -0.423 | 0.216 | 72.88 | 38.43 | beta | 3 | 3 | 0.485 | 30 | No Evidence |
|  | **Depression** | **Ratio** | **0.282** | -0.008 | 0.589 | **95.60** | 12.85 | beta | 3 | 3 | 1.634 | 30 | **Anecdotal** |
|  |  | Glutamate | 0.003 | -0.331 | 0.323 | 50.60 | 44.53 | beta | 3 | 3 | 0.400 | 30 | No Evidence |
|  |  | GABA | -0.145 | -0.457 | 0.166 | 80.60 | 31.68 | beta | 3 | 3 | 0.587 | 30 | No Evidence |
| DLPFC | **Social anxiety** | **Ratio** | **-0.282** | -0.474 | -0.061 | **99.43** | 4.98 | beta | 3 | 3 | **5.755** | 66 | **Moderate** |
|  |  | **Glutamate** | **-0.198** | -0.412 | 0.034 | **95.03** | 19.35 | beta | 3 | 3 | 1.143 | 66 | **Anecdotal** |
|  |  | **GABA** | **0.207** | -0.023 | 0.414 | **96.18** | 16.85 | beta | 3 | 3 | 1.337 | 66 | **Anecdotal** |
|  | **Trait anxiety** | **Ratio** | **-0.262** | -0.470 | -0.037 | **98.48** | 8.58 | beta | 3 | 3 | **3.371** | 66 | **Moderate** |
|  |  | Glutamate | -0.095 | -0.328 | 0.132 | 78.33 | 46.23 | beta | 3 | 3 | 0.384 | 66 | No Evidence |
|  |  | **GABA** | **0.225** | 0.007 | 0.435 | **97.63** | 12.73 | beta | 3 | 3 | 1.761 | 66 | **Anecdotal** |
|  | **Depression** | Ratio | -0.189 | -0.407 | 0.037 | 94.90 | 22.00 | beta | 3 | 3 | 1.010 | 66 | Anecdotal |
|  |  | **Glutamate** | **-0.199** | -0.445 | 0.004 | **95.78** | 19.08 | beta | 3 | 3 | 1.116 | 66 | **Anecdotal** |
|  |  | GABA | 0.098 | -0.140 | 0.311 | 78.40 | 45.35 | beta | 3 | 3 | 0.395 | 66 | No Evidence |
| IOG | **Social anxiety** | Ratio | -0.156 | -0.382 | 0.057 | 91.13 | 30.38 | beta | 3 | 3 | 0.679 | 66 | No Evidence |
|  |  | Glutamate | 0.027 | -0.188 | 0.270 | 58.73 | 59.18 | beta | 3 | 3 | 0.285 | 66 | No Evidence |
|  |  | **GABA** | **0.223** | 0.004 | 0.445 | **97.20** | 14.60 | beta | 3 | 3 | 1.668 | 66 | **Anecdotal** |
|  | Trait anxiety | Ratio | -0.044 | -0.272 | 0.189 | 64.50 | 56.80 | beta | 3 | 3 | 0.299 | 66 | No Evidence |
|  |  | Glutamate | -0.007 | -0.250 | 0.206 | 52.25 | 59.98 | beta | 3 | 3 | 0.281 | 66 | No Evidence |
|  |  | GABA | 0.005 | -0.244 | 0.230 | 51.60 | 59.50 | beta | 3 | 3 | 0.280 | 66 | No Evidence |
|  | Depression | Ratio | -0.120 | -0.342 | 0.120 | 85.23 | 40.85 | beta | 3 | 3 | 0.477 | 66 | No Evidence |
|  |  | Glutamate | -0.045 | -0.285 | 0.176 | 64.25 | 55.58 | beta | 3 | 3 | 0.302 | 66 | No Evidence |
|  |  | GABA | 0.091 | -0.145 | 0.323 | 76.95 | 47.25 | beta | 3 | 3 | 0.374 | 66 | No Evidence |

*Note.* rho is the strength of effect (range -1, 1), signed to indicate direction (positive, negative). 95% credible interval is drawn from the posterior distribution and is the range in which unobserved values can be expected to lie, given the observed data. Pd % is the estimate of the probability of direction (the signed effect, regardless of strength). Pd >95% is indicative of an existing effect. % in ROPE is the percentage of values falling in the null region, or the region of practical equivalence, for correlations this region ranges from -0.05,0.05. The smaller the percentage in ROPE the more confident inferences can be made that the posterior probabilities fall outside of is range, e.g., a measure of significance of the effect. Here, % in ROPE < 2.5 are significant. Prior (medium) refers to the prior distribution selected. Prior is bounded by use of a beta distribution and is defined by a location of 3, and scale 3. BF_10_ refers quantification of evidence in favour of the alternative hypothesis. N is the number of observations in the analysis. Category defines the degree of evidence based on BF_10_ value.
